# *Galleria mellonella* Larvae as an Infection Model to Investigate sRNA-Mediated Pathogenesis in *Staphylococcus aureus*

**DOI:** 10.3389/fcimb.2021.631710

**Published:** 2021-04-19

**Authors:** Guillaume Ménard, Astrid Rouillon, Gevorg Ghukasyan, Mathieu Emily, Brice Felden, Pierre-Yves Donnio

**Affiliations:** ^1^ Univ Rennes, CHU Rennes, INSERM, BRM [Bacterial Regulatory RNAs and Medicine], SB2H (service de Bactériologie Hygiène-Hospitalière), UMR_S 1230, F-35000, Rennes, France; ^2^ Univ Rennes, INSERM, BRM (Bacterial Regulatory RNAs and Medicine), UMR_S 1230, F-35000, Rennes, France; ^3^ Univ Rennes, CNRS, INSERM, BIOSIT (Biologie, Santé, Innovation Technologique de Rennes), UMS 3480, US_S018, F-35000, Rennes, France; ^4^ Institut Agro, CNRS, Univ Rennes, IRMAR (Institut de recherche Mathématique de Rennes), UMR 6625, F-35000, Rennes, France

**Keywords:** *Staphylococcus aureus*, sRNA, *Galleria mellonella*, virulence, regulation

## Abstract

Small regulatory RNAs (sRNAs) are key players in bacterial regulatory networks. Monitoring their expression inside living colonized or infected organisms is essential for identifying sRNA functions, but few studies have looked at sRNA expression during host infection with bacterial pathogens. Insufficient *in vivo* studies monitoring sRNA expression attest to the difficulties in collecting such data, we therefore developed a non-mammalian infection model using larval *Galleria mellonella* to analyze the roles of *Staphylococcus aureus* sRNAs during larval infection and to quickly determine possible sRNA involvement in staphylococcal virulence before proceeding to more complicated animal testing. We began by using the model to test infected larvae for immunohistochemical evidence of infection as well as host inflammatory responses over time. To monitor sRNA expression during infection, total RNAs were extracted from the larvae and invading bacteria at different time points. The expression profiles of the tested sRNAs were distinct and they fluctuated over time, with expression of both *sprD* and *sprC* increased during infection and associated with mortality, while *rnaIII* expression remained barely detectable over time. A strong correlation was observed between *sprD* expression and the mortality. To confirm these results, we used sRNA-knockout mutants to investigate sRNA involvement in *Staphylococcus* *aureus* pathogenesis, finding that the decrease in death rates is delayed when either *sprD* or *sprC* was lacking. These results demonstrate the relevance of this *G. mellonella* model for investigating the role of sRNAs as transcriptional regulators involved in staphylococcal virulence. This insect model provides a fast and easy method for monitoring sRNA (and mRNA) participation in *S. aureus* pathogenesis, and can also be used for other human bacterial pathogens.

## Introduction

The Gram-positive *Staphylococcus aureus* bacterium is a major human and animal pathogen associated with mortality and morbidity worldwide. In humans, *S. aureus* is responsible for both community-acquired and healthcare-related infections ranging from superficial to very serious or fatal diseases, including osteomyelitis, bacteremia, and endocarditis (Tong et al., 2015). *S. aureus* is also a mucosal and skin mucous commensal bacterium, colonizing about 30% of healthy individuals (van Belkum et al., 2009). The nose is the main ecological niche, and nasal carriage is a key determinant for colonization at other sites (Wertheim et al., 2005), with asymptomatic nasal carriage a major risk factor for subsequent infection (von Eiff et al., 2001).

As both a commensal and a pathogen, *S. aureus* possesses a large number of factors involved in immunomodulation and/or virulence, including adhesins, toxins, and immunomodulatory proteins that it expresses only when necessary and in a coordinated manner. The bacteria quickly adapt to various environments that change due to stresses from host immune systems or nutrient deprivation, therefore *S. aureus* survives and grows through selective modulation of its metabolism and fitness. This implies a reprogramming of its intricate network of gene regulation, triggering the expression of genes that are essential for survival in the immediate environment, and extinguishing unnecessary ones. In addition to sRNAs, two-component systems (TCS) and DNA-binding proteins regulate metabolism and virulence factor expression (Jenul and Horswill, 2018), and the *agr* quorum-sensing system is the most significant TCS in *S. aureus* (Novick, 2003). RNAIII is the effector of this *agr* system (Bronesky et al., 2016) and was also one of the first sRNAs reported in *S. aureus* (Novick et al., 1993). RNAIII is a multifunctional RNA that codes for δ-hemolysin, and it directly regulates the expression of at least 12 mRNA targets (Raina et al., 2018).

In the SRD Staphylococcal regulatory RNAs database, Sassi *et al.* listed all sRNAs identified as being expressed by various strains of *S. aureus* and other *Staphylococcaceae* (Sassi et al., 2015). The *S. aureus* sRNA targets have only been identified for a few sRNAs, mostly by MS2 tagging (Lalaouna and Massé, 2015). Among these, at least seven are involved in virulence regulation: RNAIII (Srn_3910), SprC (Srn_3610), SprD (Srn_3800), SprX (Srn_3820), RsaA (Srn_1510), Teg49 (Srn_1550), and SSR42 (Srn_4470) (Novick, 2003; Chabelskaya et al., 2010; Morrison et al., 2012; Romilly et al., 2014; Le Pabic et al., 2015; Kathirvel et al., 2016; Manna et al., 2018). Most of these pioneering studies have compared sRNA-deleted and isogenic strains with respect to animal mortality, bacterial load in infected organs, or biofilm formation. Only a few studies have monitored *in situ* expression of sRNAs (Song et al., 2012; Szafranska et al., 2014), which differ from the levels encountered in growth media. Recent publications on *S. aureus* transcriptional adaptation *in vitro* and during infection or colonization (Chaffin et al., 2012; Szafranska et al., 2014; Jenkins et al., 2015; Chaves-Moreno et al., 2016; Deng et al., 2019; Ibberson and Whiteley, 2019) have highlighted major differences between *in vitro* and *in vivo* transcriptional gene regulation. This suggests that *in situ* exploration of the regulatory networks involving sRNAs in human pathogens is essential for understanding their roles.

The purpose of this study was to gain further insights into *S. aureus* sRNA functions during infection by assessing their expression levels *in situ* in infected organisms. To do this, we used a non-mammalian *Galleria mellonella* model to investigate riboregulations at the transcriptomic level. We provide evidence that the sRNA expression patterns in infected animals differs sharply from the levels *in vitro*, with a progressive accumulation of the sRNAs SprC and SprD in the larvae up to 4 days after infection. Moreover, SprC and SprD probably act as virulence factors during larval infection, since their presence and expression lead to fewer survivors. This *G. mellonella* infection model is thus shown to be a reliable tool for investigating the riboregulations involved in bacterial virulence.

## Materials and Methods

### Strains, Media, and Genetics

All strains used are listed in **Supplementary Table 1**. For the development of the *G. mellonella* infection model, we used the *S. aureus* S75 strain obtained from a bacteremia patient. This strain is a multilocus sequence type 8 (ST8) and has a t190 *spa* type. The S75 genome was sequenced and deposited in the DDBJ/EMBL/GeneBank (accession number PRJNA273632). It has 2,728,924 base pairs, and is close to the NCTC 8325 reference strain, with 2,327 orthologous genes in common. For knock-out experiments, we used strains HG003-∆*sprC*, HG001-*ΔsprX*, HG001-∆*sprD*, and HG003-*ΔrnaIII.* These were constructed in our lab in HG001 and HG003 backgrounds, and both are derivatives of NCTC 8325 (Herbert et al., 2010). For *rnaIII* overexpression, we used a QIAprep spin miniprep kit (Quiagen) on the *S. aureus* Newman strain to extract the vector and transfer it to HG003 to create pRMC3-*rnaIII*. All strains were grown in Luria-Bertani broth (LB) at 150 rpm at 37°C, and in the presence of 10 µg/ml chloramphenicol for HG003-pRMC3-*rnaIII*. For larval infection, overnight cultures were centrifuged at 3,000 rpm for 10 minutes then washed twice with sterile PBS. The inocula were prepared in sterile PBS by OD adjustment, verified by plating on tryptic soy agar after serial dilutions, then incubated for 24 hours at 37°C.

### *Galleria mellonella* Infection Model

The *G. mellonella* larvae were purchased from Sud-Est Appats (Queige, France). They were stored in the dark at 4°C, and used within 7 days of delivery. To standardize the experiments, we selected larvae weighting about 250-300 mg and without color alteration. They were incubated at room temperature for 2 hours before injection, then disinfected externally with 70% ethanol. The infection was carried out using a KDS 100 automated syringe pump (KD Scientific) and a 300 µl Hamilton syringe. Haemocoel was injected with 10 μl of the S75 strain at 10^6^, 10^7^, 10^8^, or 10^9^ CFU/ml. The infected larvae were placed on Petri dishes and incubated at 37°C. Mortality was monitored daily for 6 days, and larvae were considered dead when they were immobile, no longer responding to stimuli, and melanized. For each condition, 3 independent experiments were performed using 10 infected larvae. The controls were uninjected larvae and larvae injected with 10 µl of sterile PBS. For the experiments using sRNA-deleted isogenic strains, either HG003 or HG001 was used as the control. For *rnaIII* overexpression experiments, pRMC3-*rnaIII* stability was assessed by randomly plating selected colonies grown from fat-body homogenates on agar containing 10 µg/ml of chloramphenicol at different time points after infection.

### Bacterial Growth in the Larvae

One hundred larvae were infected with 10^6^ CFU *S. aureus* S75. At different times post-infection, 3 living larvae were randomly selected, disinfected with 70% ethanol, dried, and ice-chilled for 10 minutes. The hemolymph and fat bodies were separated from each larva and pooled. Briefly, after an incision with a scalpel, the larvae were squeezed to collect hemolymph, and pooled into a microcentrifuge tube collection. On average, 15 to 40 µl of hemolymph per larva were collected. The remaining fat bodies were mechanically homogenized with sterile distilled water at 4°C. These collections were diluted with sterile distilled water, plated on Baird-Parker agar, and incubated for 48 hours at 37°C. The bacterial loads were estimated by counting the colony-forming units in each larva at each time point. The *in vivo* growth curve was monitored every day for 6 days. To investigate early growth, the same experiment was performed but this time just observing the fat bodies over 24 hours, plating every 4 hours. For each condition, 3 independent experiments were performed.

### Immunohistochemistry

We challenged 50 larvae with 10^6^ CFU S75, and 2 living larvae were randomly selected at various times post-infection. Infected and uninfected larvae were fixed for 2 weeks in 4% neutral buffered formalin. For efficient embedding, the larvae were sectioned longitudinally before being placed into paraffin and processed in a Shandon Excelsior ES (Thermo Scientific). The paraffin-embedded tissues were cut into 4-µm sections using a Microm HM340E microtome with section transfer system (Thermo Fisher). These sections were mounted on positively charged SuperFrost Plus slides (VWR) then dried for 60 minutes at 58°C. Immunohistochemical staining was performed using a Ventana purple-detection kit and their DISCOVERY ULTRA automated stainer. Following dewaxing with EZ prep solution (Ventana) at 75°C for 8 minutes, endogenous peroxidase was blocked for 12 minutes at 37°C with DISCOVERY Inhibitor (Ventana). After rinsing, the slides were incubated for 60 minutes at 37°C with Bio-Rad rabbit polyclonal anti-*S. aureus* antibodies (0300-0084). Signal enhancement and detection were performed using the DISCOVERY purple kit. Slides were counterstained for 16 minutes with Ventana hematoxylin II (790-2208) and for 4 minutes with Ventana bluing reagent (760-2037). They were then rinsed, manually dehydrated, and cover-slipped. Image acquisition was done with NanoZoomer (Hamamastu Photonics) and analyzed using the accompanying NDP.view2 software.

### Bacterial Isolation and RNA Extraction

The protocol for differential lysis was adapted from Robbe-Saule et al. (2017), Two living larvae were randomly selected, externally disinfected with 70% ethanol, dried, then placed into Petri dishes. Using sterile forceps holding the head of the larvae, the larval contents were extracted by applying pressure using a 10 ml syringe plunger. This step allowed the cuticle and the larval contents to be correctly separated. After removing their cuticles, larval contents were mechanically homogenized in 5 ml of sterile PBS and centrifuged at 2,500 rpm for 5 minutes. The supernatant was recovered then combined with 50 mM calcium chloride and 1 ml Proteinase K solution (12 mg/ml) supplemented with 1 M tris(hydroxylmethyl)aminomethane hydrochloride (pH 8). This mix was incubated for 30 minutes at 50°C, then centrifuged at 13,000 rpm for 20 minutes at 4°C. For total bacteria RNA extractions, pellets were suspended in 500 µl of lysis buffer (20 mM sodium acetate, 1 mM EDTA, 0.5% SDS pH 5.5) and transferred into 1.5 ml RNase-free centrifuge tubes containing 500 µl of zirconium beads and 500 µl of phenol (pH 4). Cells were broken up in a FastPrep FP120 cell disruptor (MP Biomedicals). RNA was extracted by the phenol-chloroform method. The top clear layer was removed and RNA was precipitated overnight at -80°C in isopropanol supplemented with 0.3 M sodium acetate and glycogen. Finally, RNAs were pelleted by centrifugation at 13,000 rpm for 30 minutes at 4°C. The pellets were then cleaned with 70% ethanol, dried, and dissolved in RNase-free water. To eliminate DNA contamination, samples were processed with the TURBO DNA*-free* kit (Thermo Fisher) as per the manufacturer’s recommendations. A second precipitation was performed to remove putative contamination, with RNAs treated with 100% ethanol supplemented with 0.3 M sodium acetate and glycogen. RNA quality and quantity were assessed using a NanoDrop spectrophotometer (Thermo Fisher) and agarose gel electrophoresis.

We used proteinase K to create a differential lysis method that would preferentially degrade larval tissue, and we verified its efficiency through method development. Briefly, we first controlled that proteinase K did not significantly damage bacterial cells by comparing numbers of CFU of *S. aureus* before and after incubation with proteinase K *in vitro*. Then, we applied this method to RNA extraction, and we assessed the integrity of RNAs. Finally, we tested increasing concentrations of proteinase K on infected larvae.

Bacterial RNA extraction was also performed from *in vitro* cultures with a Proteinase K solution at both the mid-exponential and stationary phases in LB broth. At designated time *in vitro* bacteria cultures were incubated for 30 minutes at 50°C with a proteinase K solution, then centrifuged at 13,000 rpm for 10 minutes at 4°C. From the pellet, total RNA was extracted according to the protocol described above.

### Monitoring RNA Expression Levels

We challenged 100 larvae with 10^6^ CFU S75, and quantitative reverse transcription PCR (qRT-PCR) was used to monitor sRNA expression during larval infection. We analyzed RNAIII, SprC, SprD, SprX, and RsaA, sRNAs present and expressed in the S75 strain (Bordeau et al., 2016), as well as the mRNA controls GyrB and SigA. All primers used are listed in **Supplementary Table 2**. RNA samples were reverse-transcribed and amplified using a high-capacity cDNA reverse transcription kit and the PowerUp SYBR green master mix (Thermo Fisher) as recommended by the manufacturer. Reverse transcription was performed using 0.3 µg of RNA in 10 µl of nuclease free water, and 10 µl of the 2X RT master mix. The reverse transcription run followed the manufacturer’s recommendations. Resulting cDNA were stored at -20° C until qPCR. For qPCR, the reaction volume was 20 µl comprising 10 µl SYBR Green 10X master mix, 5 µl cDNA diluted 1/100, forward and reverse primers at a final concentration of 500 nM, completed with nuclease free water. The reaction was performed on the Applied Biosystems 7500 Real-Time PCR System using the standard mode.

Two different methods were used for data interpretation: comparison against the *in vitro* RNA expression levels of S75 at the exponential and stationary growth phases; and comparison of sRNA transcript levels in the larvae at an early (12-hour) post-infection step against the levels at various post-infection times. sRNA expression was normalized against housekeeping mRNA transcripts, and their relative expression levels were inferred using the ΔΔCt method. All experiments were performed in triplicate.

### Statistical Analysis

*G. mellonella* survival profiles were determined using the log-rank test and Kaplan-Meier survival plots. For daily mortality comparisons, a Student’s *t-*test was used to compare the expressions in sRNA-deleted and isogenic strains. Sample *t*-tests were used for comparing the sRNA profiles between experiments, for instance *in vivo* versus *in vitro* expression during the exponential or stationary growth phases. To monitor the trend in larval sRNA expressions at each selected post-infection time point, we analyzed the variance and did multiple comparisons of means using Tukey contrasts. A two-sample test was used to analyze sRNA expression in the larvae during the first 24 hours after infection, then up to 96 hours later. Larval sRNA expression and risk-adjusted mortality rates were compared using Spearman’s correlation coefficient. The risk-adjusted mortality corresponded to the ratio of the number of dead larvae to the number of larvae still alive at the time of the experiment. Results were considered statistically significant when *p* < 0.05.

## Results

### Setting up a *G. mellonella* Infection Model

**Figure 1:**
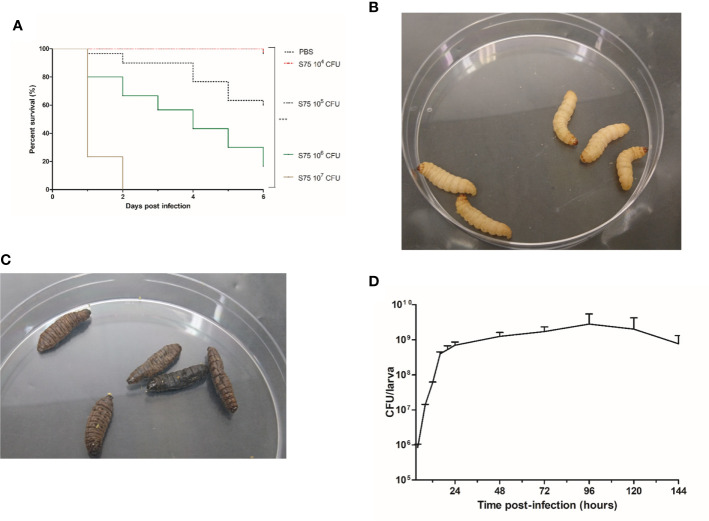
Kaplan-Meier survival plots and bacterial levels in infected *Galleria mellonella* larvae infected with *Staphylococcus aureus*. **(A)** Survival of *G. mellonella* larvae after inoculation with 10^4^ to 10^7^ CFU of the *S. aureus* S75 strain. Plots show an average of 3 independent experiments with 10 larvae per group (N= 150), with mortality monitored daily for 6 days. PBS-injected larvae were the negative control. Significant differences were defined as ***p < 0.001. **(B)** *G. mellonella* larvae on day 6 after being challenged with 10 µl of PBS. **(C)** Dead *G. mellonella* larvae at day 6 after infection with 10^6^ CFU of *S. aureus* S75. **(D)** Time evolution of bacterial burden in homogenized cuticle-free larvae infected by 10^6^ CFU of *S. aureus* S75. Data are the sum of 3 independent experiments, with 100 larvae per experiment (N = 300). Error bars represent the standard deviations (SD).

To determine larval susceptibility to infection with *S. aureus* S75, we injected them with different inocula and monitored mortality daily. We observed that survival rates in *G. mellonella* were dependent on the S75 dosage (**Figure 1A**). No deaths or melanization were detected either in the control group or after injecting 10^4^ CFU of S75 (**Figure 1B**). However, all larvae died within 48 hours after injection with 10^7^ CFU. At day 6, mortality was 40% and 80% after injection with 10^5^ or 10^6^ CFU, respectively. Dead larvae were immobile as well as black due to melanization (**Figure 1C**). For our model, we selected a dose of 10^6^ CFU, as a gradual reduction in larval survival rates occurred over the 6-day experiments with that amount.

To determine S75 growth within the larvae, they were infected by 10^6^ CFU, then 3 were sacrificed at different times post-infection to count the *S. aureus* bacteria present in the hemolymph and fat bodies. In the fat body, bacterial growth was biphasic, with a fast increase from 10^6^ to 10^9^ CFU/larva up to 24 hours post-infection, and a steady load of about 10^9^ CFU/larva from day 1 to day 6 post-infection (**Figure 1D**). Growth was different in the hemolymph, with bacterial loads increasing up to 72 hours but not exceeding 6.10^6^ CFU/larva (**Supplementary Figure 1**).

### Monitoring *S. aureus* Infection of Larvae With Immunohistochemical Staining

**Figure 2:**
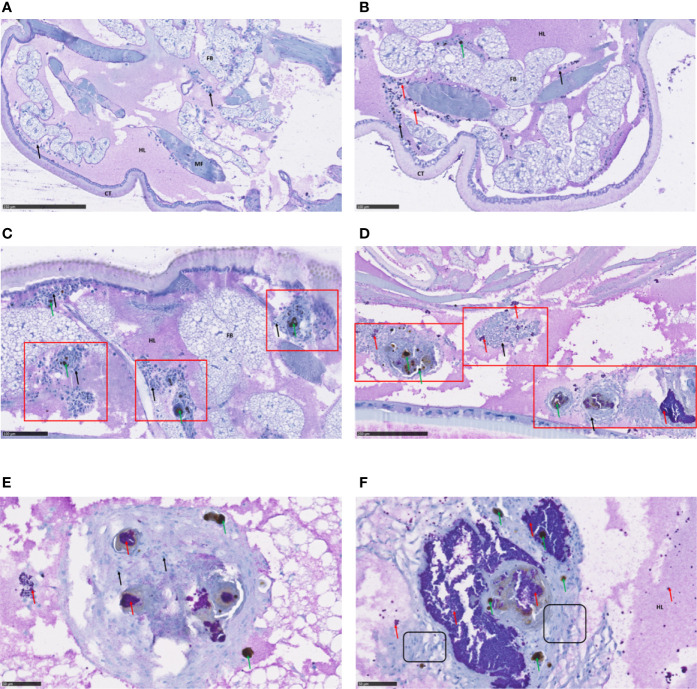
Immunohistochemical analysis of *Galleria mellonella* larva sections. Immunohistochemical analysis and hematoxylin staining were performed to visualize bacteria within the infected host and to examine histological sections. *G. mellonella* larvae were challenged with 10^6^ CFU *S. aureus* S75, then collected at fixed times and examined. Shown are: **(A)** non-infected larvae; **(B, C)** infected larvae 24 hours after infection, showing evidence of immune activation; **(D)** infected larvae at 72 hours, with numerous nodules in the presence of hemocytes and bacteria; and **(E, F)** higher-magnification views of pigmented nodules at 96 hours after infection. *S. aureus* bacteria appeared in purple and are indicated with red arrows, hemocytes were stained in blue, with a deep blue nucleus and are indicated by black arrows, melanization (black spots) by green arrows, nodule clusters are outlined in red squares, and hemocytes of different morphology framed in black boxes. Scale bars are indicated on each picture. Key: CT, cuticle; HL, hemolymph; FB, fat body; MF, muscle fibers.

We used immunohistochemical staining and analysis to monitor infection with *S. aureus* S75 as well as its impact on various larval tissues up to 4 days after infection (**Figure 2**). In the control larvae, the hemocyte immune cells were subcuticular, scattered in the fat body and around the digestive tract (**Figure 2A**). We observed an immune response in the infected larvae as early as 1 day post-infection, with hemocyte recruitment around the bacteria, which are clustered into ‘grape-like’ shapes (**Figure 2B**). Bacteria often co-localize with host immune-cells and form nodules with each other (**Figure 2C**). Infection foci spread progressively, affecting the entire larval body, including its nervous system (**Supplementary Figure 2**). During infection, the immune response increased, with a predominance of pigmented nodules reflecting melanization (**Figure 2D**). Nodules were composed of bacteria, melanin, as well as numerous hemocytes, and we could visualize morphologically distinct immune cells (**Figures 2E, F**).

### Monitoring *S. aureus* sRNA Expression During Infection

The challenge was to extract enough intact bacterial RNA for qRT-PCR assays of their expression levels. To create a differential lysis method which would only degrade larval tissues, we used Proteinase K. We began by showing that this serine protease does not damage bacterial cells, since the *S. aureus* CFU counts were not statistically different with or without Proteinase K (**Supplementary Figure 3A**). We also saw that Proteinase K did not influence the quality of the bacterial RNA (**Supplementary Figure 3B**). We were thus able to extract intact bacterial RNA from the mixture of eukaryotic and prokaryotic cells, and as expected, we observed an increased ratio of bacterial to larval RNA (**Supplementary Figure 3C**). Therefore, increasing concentrations of Proteinase K improved detection of *S. aureus* targets, and this was shown by the lowered cycle thresholds for the housekeeping genes *gyrB* and *sigA* (**Supplementary Table 3**). Finally, we extracted bacterial RNA from the whole infected larvae, at time points between 12 and 96 hours after infection.

**Figure 3:**
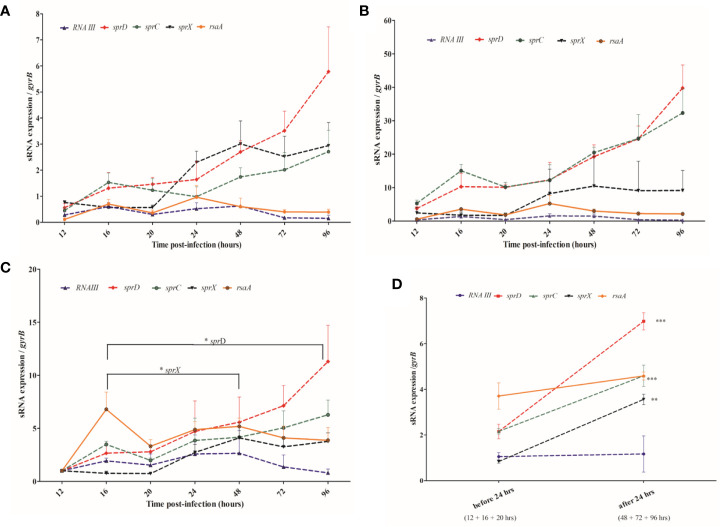
Monitoring sRNA expression from 12 to 96 hours after *Staphylococcus aureus* infection of larva **(A, B)** sRNA expression levels in the *Galleria mellonella* larvae compared with the levels found at the stationary **(A)** and exponential **(B)** growth phases in liquid media. **(C)** sRNA expression levels in the larvae at various time points are compared to their values 12 hours after infection. **(D)** Comparison of pooled sRNA expression in the larvae before and after the 24-hour post-infection point. sRNA expression monitored by qRT-PCR was normalized against GyrB mRNA, and 3 independent experiments were realized for each condition, with 100 larvae per experiment (N= 300). Error bars represent the SDs. Significant differences were defined as *p < 0.05; **p < 0.01; and ***p < 0.001.

We assessed *S. aureus* sRNA transcript levels *in situ* at various times post-infection and compared these to those seen at the exponential and stationary levels in culture media (**Figures 3A, B**). We found that *rnaIII* and *rsaA* expression levels were relatively stable across the 4-day infection of the larvae. Interestingly, those of *sprC*, *sprD*, and *sprX* progressively increased during infection, but the expression levels of these sRNAs in the larvae were dependent upon the *in vitro* ‘comparator’ set and the phase examined. Indeed, ratios ranged from 2-6 when stationary phase cultures were used as a control, and from 10-40 when selecting the exponential phase cultures. In addition, whereas the *in vivo* gene expressions of *sprD*, *sprC*, and *sprX* were higher than both control conditions (respectively *p* < 0.0001, 0.0001, and 0.001 when compared with the *in vitro* exponential phase, and *p* = 0.0005, 0.003 and 0.001 when compared with the stationary phase), *rsaA* was more expressed *in vivo* than in the *in vitro* exponential phase (*p* = 0.001), but less expressed *in vivo* than the *in vitro* stationary phase (*p* = 0.0001). Such differences highlight the difficulty of choosing a good *in vitro* comparator. It is noteworthy that no significant difference was observed with *rnaIII*.

Since it was difficult to compare the *in vitro* and *in vivo* sRNA expression levels, we compared *in vivo* sRNA expression to the first qRT-PCR value obtained at the beginning of infection, 12 hours after injection (**Figure 3C**). We saw a trend for *sprC* and *sprD*, which were both upregulated and had peaks 96 hour after injection. While *sprD* was significantly upregulated 16-96 hours post-infection (*p* = 0.043), the increase observed for *sprC* was not statistically significant. *sprX* was increased early, with a peak 48 hours after infection, and its expression was significantly higher at that point than at 16 hours (*p* = 0.029). *rnaIII* was weakly expressed at all-time points, and none of the observed variations were significant. No significant variations were observed for *rsaA*.

sRNA expression levels were examined in the periods before (16-20 hours) and after (48-96 hours) the 24-hour post-infection point (**Figure 3D**). Higher levels of *sprC*, *sprD*, and *sprX* were found after the first day of infection (*p* = 0.0001, *p* < 0.0001, and *p* = 0.0007, respectively), while *rnaIII* and *rsaA* expression levels stayed relatively even.

### sRNAs Are Involved in *G. mellonella* Virulence

**Figure 4:**
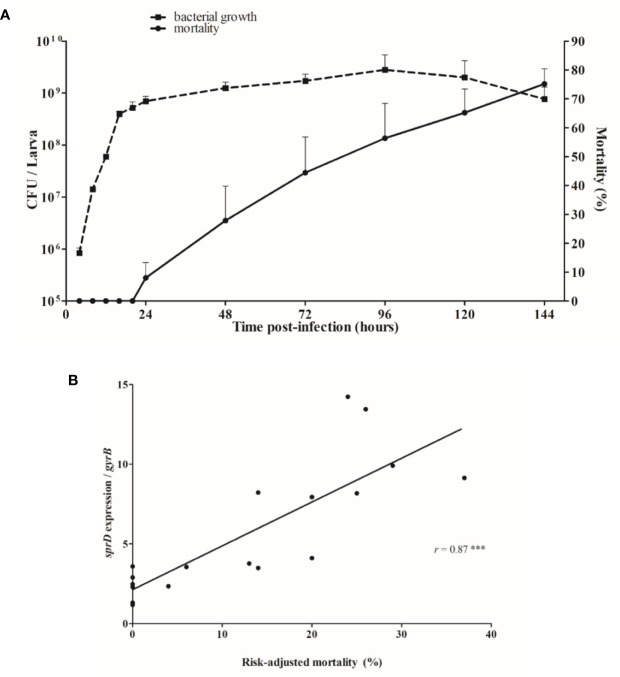
Relationship between mortality and sRNA expression levels. **(A)** Correlation between growth of *Staphylococcus aureus* (CFU/larva) and the mortality of infected *Galleria mellonella* larvae. **(B)** Correlation between *sprD* expression and risk-adjusted mortality. The *in vivo* transcript levels of sprD were calculated to those for *gyrB* mRNA and compared to the expression obtained 12 hours after infection. Each point represents relative *sprD* expression during infection associated with the risk-adjusted mortality at the same time point, and corresponds to the sum of 3 independent experiments, with 100 larvae per experiment (N= 300). Key: *r*, Sperman’s coefficient; ***p < 0.001.

Larvae mortality started 1 day after infection, when the bacterial load had reached about 10^9^ bacteria per larva, and death increased continuously thereafter (**Figure 4A**). Since time post-infection and mortality are linked, to minimize interpretation bias, we looked at how sRNA levels corresponded to the risk-adjusted mortality. In fact, *sprD* expression levels are strongly correlated to the risk-adjusted mortality (*r* = 0.87, *p* < 0.0001), implying that the more SprD is expressed, the more virulent the strain (**Figure 4B**).

To look for connections between sRNA expression and virulence, we compared the mortality induced by sRNA-deletion strains to that of isogenic controls. We examined S75 and two another strains, HG001 or HG003, both of which are derived from the NCTC 8325 reference strain that is phylogenetically related to S75. We did not observe any differences in larval mortality in these three ST8 strains (**Supplementary Figure 4**).

**Figure 5:**
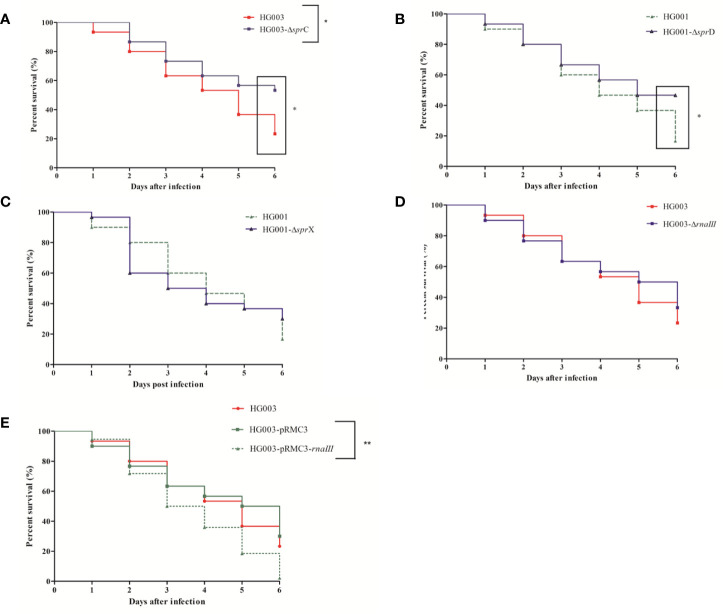
Kaplan-Meier survival plots of *Galleria mellonella* larvae infected with various forms of *Staphyllococcus aureus*. Larvae were injected with 10^6^ CFU of wild-type HG001 or HG003, three types of isogenic sRNA mutants, or with a strain overexpressing *rnaIII*. PBS-injected larvae were used as negative controls. Shown are the survival rates over 6 days for the following: **(A)** HG003 and its HG003*-ΔsprC* isogenic mutant; **(B)** HG001 and HG001*-ΔsprD*; **(C)** HG001 and HG001-*ΔsprX*; **(D)** HG003 and HG003*-ΔrnaIII*; and **(E)** HG003 compared to both HG003-pRMC3 and HG003-pRMC3-*rnaIII*. Significant differences were defined as *p < 0.05; **p < 0.01. Data are shown from 3 independent experiments using 10 larvae per group for each condition.

At all tested times, larval mortality was significantly reduced (*p* = 0.03) in comparison with the control after infection with HG003-∆*sprC* (**Figure 5A**). However, only late mortality (at day 6), was significantly reduced (*p* = 0.01) in HG001-*ΔsprD* infection as compared to the isogenic strain (**Figure 5B**). In contrast, no significant differences (*p* = 0.70 and *p* = 0.64, respectively) were seen after infection with HG001-*ΔsprX* or HG003-*ΔrnaIII* (**Figures 5C, D**).

Since *rnaIII* expression in the larvae was very low, we wondered whether overexpressing *rnaIII* would affect virulence. We found that *rnaIII* overexpression significantly increased mortality (*p* = 0.007) (**Figure 5E**), with enhanced larval necrosis and a complete destruction of larval tissue (**Supplementary Figure 5**). High *rnaIII* expression thus seems to be detrimental during larval infection.

## Discussion

Here, we present a *G. mellonella* model set up to study the impacts of *S. aureus* riboregulations during infection. Indeed, accumulating evidence has shown the importance of numerous sRNAs in bacterial pathogenicity (Novick, 2003; Chabelskaya et al., 2010; Morrison et al., 2012; Romilly et al., 2014; Le Pabic et al., 2015; Kathirvel et al., 2016; Manna et al., 2018). The larvae of this greater wax moth have been extensively used to study the virulence of many microorganisms (Tsai et al., 2016) and for testing the efficacy of antimicrobial compounds (Cutuli et al., 2019) for several reasons: costs are low; no specific training or equipment is required; and many larvae can be infected simultaneously. In addition, invertebrates do not possess nociceptors and are thus not sensitive to pain, so there are no restrictive ethical rules as exist for vertebrate use (Bismuth et al., 2019). *G. mellonella* lacks adaptive immunity, but the organism develops an innate immune response that is similar to that of vertebrates, including cellular and humoral responses which are responsible for nodulation and phagocytosis (Pereira et al., 2018). Unlike *Caenorhabditis elegans* and *Drosophila melanogaster*, infections can be performed at 37°C in *G. mellonella*, which is an advantage for studying the bacterial virulence of *S. aureus* or other human pathogens. *G. mellonella* infections can be caused either *via* ingestion or by intrahemocoelic injection, and the latter method allows for close control of the inoculum (Ramarao et al., 2012). In most studies, the model has been used to examine the antimicrobial activity of drugs or to screen for *S. aureus* virulence factors (Quiblier et al., 2013; Ferro et al., 2016; Silva et al., 2017). A previous work showed that *G. mellonella* could also be used for other purposes, especially for examining *S. aureus* interaction with the larval immune response and for how the bacteria induces the expression of immune-related peptides (Sheehan et al., 2019). We therefore used this model to investigate riboregulations in staphylococcal virulence. As proof of concept, we applied it to examine sRNAs already established as having major impacts on virulence.

Consistent with a recent study (Sheehan et al., 2019), we saw an early bacterial multiplication that triggered an immune response in the form of hemocyte recruitment. Majority of hemocytes are phagocytic cells similar to vertebrate neutrophils which are involved in abscess formation (Kobayashi et al., 2015). During *S. aureus* infection, we visualized nodules which could correspond to abscesses in vertebrate tissues. The number of nodules increased during infection as well as being spread throughout the entire larva, implying systemic infection. During human infection, circulating bacteria are scarce (Opota et al., 2015), as they are rapidly killed by phagocytes. The surviving bacteria migrate, accumulating in host organs and causing tissue damages and/or abscesses. Similarly, in infected larvae, the bacterial load is higher than in the fat body compared to the hemolymph which is the larval equivalent to the bloodstream.

We investigated how staphylococcal sRNAs were expressed during infection. The ‘proof of concept’ was achieved after we examined a subset of five sRNAs: RsaA and RNAIII which are both expressed from the core genome; and SprC, SprD, and SprX from pathogenicity islands. Indeed, with the exception of RNAIII, the expression levels of each of these sRNAs in the larvae differed from what can be observed in liquid cultures during the exponential or stationary growth phases. Therefore, *in vitro* sRNA expression does not mimic that of *in vivo* conditions, a difference already observed for other staphylococcal sRNAs isolated from nasal carriers, abscesses, cystic fibrosis patients, and mouse-model bone infections (Song et al., 2012; Szafranska et al., 2014). Frequently, the *in vivo* expression of these sRNAs has been arbitrarily compared to *in vitro* exponential or stationary growth phases, and it is therefore difficult to determine the precise evolution of staphylococcal sRNAs during disease (Song et al., 2012; Szafranska et al., 2014). Our observations agree with this, proving the importance of the calibrator used for analyzing the results. To compare bacterial colonization at different stages of infection, the *in vivo* gene expression of *S. aureus* was previously monitored (without an *in vitro* calibrator) in mice and cotton rats presenting with nasal colonization, bacteremia, or heart lesions (Jenkins et al., 2015). We therefore decided to apply this method to the examination of *in vivo* sRNA expression. In our model, we measured sRNA expression 12 to 96 hours after infection, setting the initial 12-hour value as our reference. We found that the infection stage influences sRNA expression. Variations in the expression levels of *sprD* and *sprX*, as well as their accumulation in bacterial cells throughout the infection, suggest that these sRNAs both contribute to infection. It also indicates that sRNAs are not expressed continuously, and that they are tightly regulated at the different stages of infection.

Larval mortality began at 24 hours after infection, and increased progressively. There is no link between bacterial growth and mortality, as the bacterial load remained stable between days 1 and 6. *In vitro*, the *agr* quorum-sensing (QS) system senses population density, promoting the expression of secreted proteins and inhibiting adhesins *via* RNAIII regulation (Novick, 2003). This system does not seem to be activated during larval infection, since the post-infection expression of *rnaIII* remains low. These results were further supported by the lack of significant differences we observed in larval mortality between HG003 and the HG003-∆*rnaIII* mutant. RNAIII does not affect *S. aureus* survival on fruit flies (Garcia-Lara et al., 2005), suggesting that RNAIII might not play a key role in virulence in invertebrates. However, we showed that increasing *rnaIII* expression actually promotes mortality, with efficient larval necrosis. Furthermore, we found low *rnaIII* expression levels in the infected larvae, just as low amounts were detected in a mouse model of osteomyelitis, in human abscesses, during murine vaginal colonization, and in the lungs of cystic fibrosis patients (Song et al., 2012; Szafranska et al., 2014; Deng et al., 2019; Ibberson et al., 2019). Nevertheless, this sRNA is still involved in virulence since disruption of the *agr* QS system inhibits the upregulation of many toxins and proteases and a *ΔagrB* strain protects mice from mortality (Date et al., 2014). A Δ*rnaIII* strain was also shown to attenuate virulence in a murine intracranial abscess model (Gong et al., 2014). Numerous studies have reported *agr* downregulation in multiple human host niches responding to different environmental cues (Dastgheyb and Otto, 2015). Indeed, environmental cues modify the behavior of *S. aureus* and counter its *agr* system, which is sensitive to many stresses or host-derived factors such as the reactive oxygen species that are produced in infected larvae by activated hemocytes (Tsai et al., 2016; Jenul and Horswill, 2018). This could prevent *agr* activation, explaining the low expression of *rnaIII*, and thereby counterbalance *S. aureus* virulence. In contrast, *rnaIII* overexpression probably overwhelms the host immune response, which would explain the resulting virulence enhancement. Further studies are needed to clarify the central role of RNAIII in virulence regulation.

Staphylococcal virulence is also affected by others sRNAs: SprC (Le Pabic et al., 2015), SprD (Chabelskaya et al., 2010), and SprX (Kathirvel et al., 2016). The expression levels of *sprC* and *sprD* are increased when the bacteria are in contact with human serum (Carroll et al., 2016). However, little monitoring of their expression levels during animal infection has been done, so this was one objective of our study. For both SprC and SprD, expression increased progressively in infected larvae, and we observed decreased larval mortality with the Δ*sprC* strain. Since SprC was previously shown to attenuate staphylococcal virulence in a mouse infection model (Le Pabic et al., 2015), our data are somewhat surprising, indicating that results might vary depending on the animal model and strains used. Results of Le Pabic et al. were obtained using a Newman strain which among other changes differs with a mutation in saeS (Herbert et al., 2010). This mutation induces a particular exoprotein profile by comparison with HG003. Similarly, the survival profile in a sepsis mouse model was not strictly superposable between these two strains (Herbert et al., 2010). These differences may explain these inconsistent results. A strong positive correlation between *sprD* expression and risk-adjusted mortality was also observed. These preliminary results need to be confirmed in a mammalian model and in humans. *S. aureus* bacteremia is a leading cause of morbidity and mortality (Yilmaz et al., 2016), yet not enough relevant biomarkers have been identified for reliable prediction of clinical outcomes. The monitoring of *sprD* expression alone could be a potential biomarker of severity, and our observations have confirmed that SprD plays a major role in bacterial virulence in two different animal models of infection (Chabelskaya et al., 2010). SprD has one identified target, Sbi, which is an immune-evasion molecule acting at the interface between the innate and adaptive immune systems (Smith et al., 2011), and which is downregulated by SprD (Chabelskaya et al., 2010). There is no specific adaptive system in the greater wax moth, nevertheless, the staphylococcal Sbi was shown to be important in the *Galleria* model since the mortality was attenuated with a Δ*sbi* USA300 strain (Zhao et al., 2020). So it is likely that SprD acts on other molecular targets to influence staphylococcal virulence.

Our study confirms the importance of sRNAs in *S. aureus* infection in animals, and the handy *Galleria mellonella* infection model can easily be extended to the investigation of all 50 bona fide sRNAs known to be expressed by *S. aureus* (Liu et al., 2018). sRNAs belong to complex gene regulatory networks (Fechter et al., 2014), and testing their involvement in virulence requires an understanding of their connections and interactions within the entire regulon. Our next step will be to perform a transcriptomic analysis to compare sRNA-deletion and isogenic strains in order to obtain a more comprehensive view of their participation in virulence.

## Data Availability Statement

The datasets presented in this study can be found in online repositories. The names of the repository/repositories and accession number(s) can be found in the article/**Supplementary Material**.

## Author Contributions

Conceptualization: GM and P-YD. Experiments: GM and AR. Microscopy: GG and GM. Statistics: ME and GM. Writing – original draft: GM. Writing – review and editing: GM, AR, BF, and P-YD. All authors contributed to the article and approved the submitted version. 

## Conflict of Interest

The authors declare that the research was conducted in the absence of any commercial or financial relationships that could be constructed as a potential conflict of interest.

## Supplementary Material

Supplementary Figure 1 Post-infection bacterial burden in G. mellonella. Graph showing S. *aureus* S75 bacterial burden over time in the hemolymph of larvae infected by 10^6^ CFU. Data are the result of 3 independent experiments, each using 100 larvae per group. Error bars represent the SDs.

Supplementary Figure 2 Cross-sectional analysis for monitoring infection in the larvae. **(A)** PBS-injected larvae, the negative controls. **(B)** Larvae were injected with 10^6^ CFU of *S. aureus* S75, and are shown here 96 hours later. Each black ellipse represents a nodule showing melanization, and the nodules are scattered around the entire larvae, including within the nervous system (NS).

Supplementary Figure 3 Effect of Proteinase K on total RNA extractions. **(A)** Bacteria in the stationary phase were treated with 3 mg/ml Proteinase K (proK) or PBS for 30 minutes at 50°C. Results are the means of 3 independent experiments and are expressed in CFU/ml. **(B)** Total bacterial RNA counts after agarose gel electrophoresis and ethidium bromide exposure with (+) or without (-) a 3mg/ml Proteinase K solution. Bacterial RNA was extracted during the stationary phase after overnight culture. **(C)** Total RNA extraction of *G. mellonella* infected with 10^8^ CFU of *S. aureus* S75. For each condition, 5 larvae were infected, and 2 were randomly chosen for RNA extraction (N= 6). RNA extraction was performed 30 minutes after injection, and several concentrations of Proteinase K were applied. For each condition, after DNAse process, total RNA quality and quantity were assessed using a NanoDrop spectrophotometer and 0.5% agarose gel electrophoresis.

Supplementary Figure 4 Comparison of Kaplan-Meier survival plots of larvae infected with ST8 strains. Survival of *G. mellonella* larvae infected with 10^6^ CFU of the *S. aureus* strains S75, HG003, and HG001. Mortality was monitored for 6 days. The plot is the result of 3 independent experiments using 10 larvae per group, and PBS-injected larvae were used as a negative control.

Supplementary Figure 5 RNAIII overexpression and macroscopic effects on infected *G. mellonella larvae*. Larvae were inoculated with 10^6^ CFU and mortality monitored for 6 days, at which point dead larvae were observed. **(A)** Dead and melanized larvae after HG003-pRMC3 infection. **(B)** Larval necrosis was increased after infection with HG003-pRMC3-*rnaIII*.

Supplementary Table 1 Strains and vectors used for *G. mellonella* infection.

Supplementary Table 2 Primers used for qRTPCR.

Supplementary Table 3 Proteinase K Effects on the cycle thresholds of *S. aureus* housekeeping genes observed in qRTPCR.
