# Supplementary figures and images for: Galleria mellonella Larvae as an Infection Model to Investigate sRNA-Mediated Pathogenesis in Staphylococcus aureus
Source: Front Cell Infect Microbiol. 2021 Apr 19;11:631710. doi: 10.3389/fcimb.2021.631710 (PMC8089379; doi:10.3389/fcimb.2021.631710)

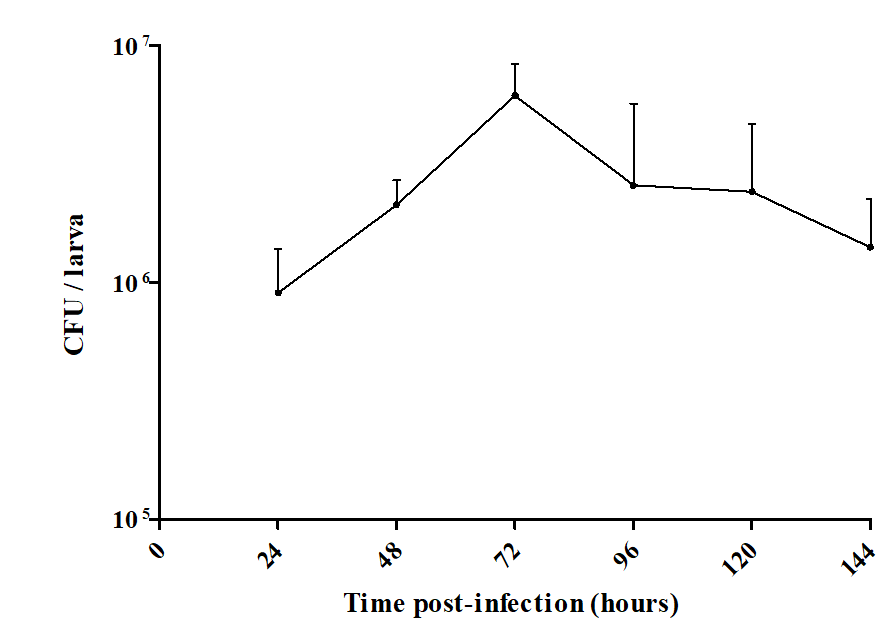

Supplement: Supplementary Figure 1 — Post-infection bacterial burden in G. mellonella. Graph showing S. aureus S75 bacterial burden over time in the hemolymph of larvae infected by 106 CFU. Data are the result of 3 independent experiments, each using 100 larvae per group. Error bars represent the SDs. [file Image_1.tif]

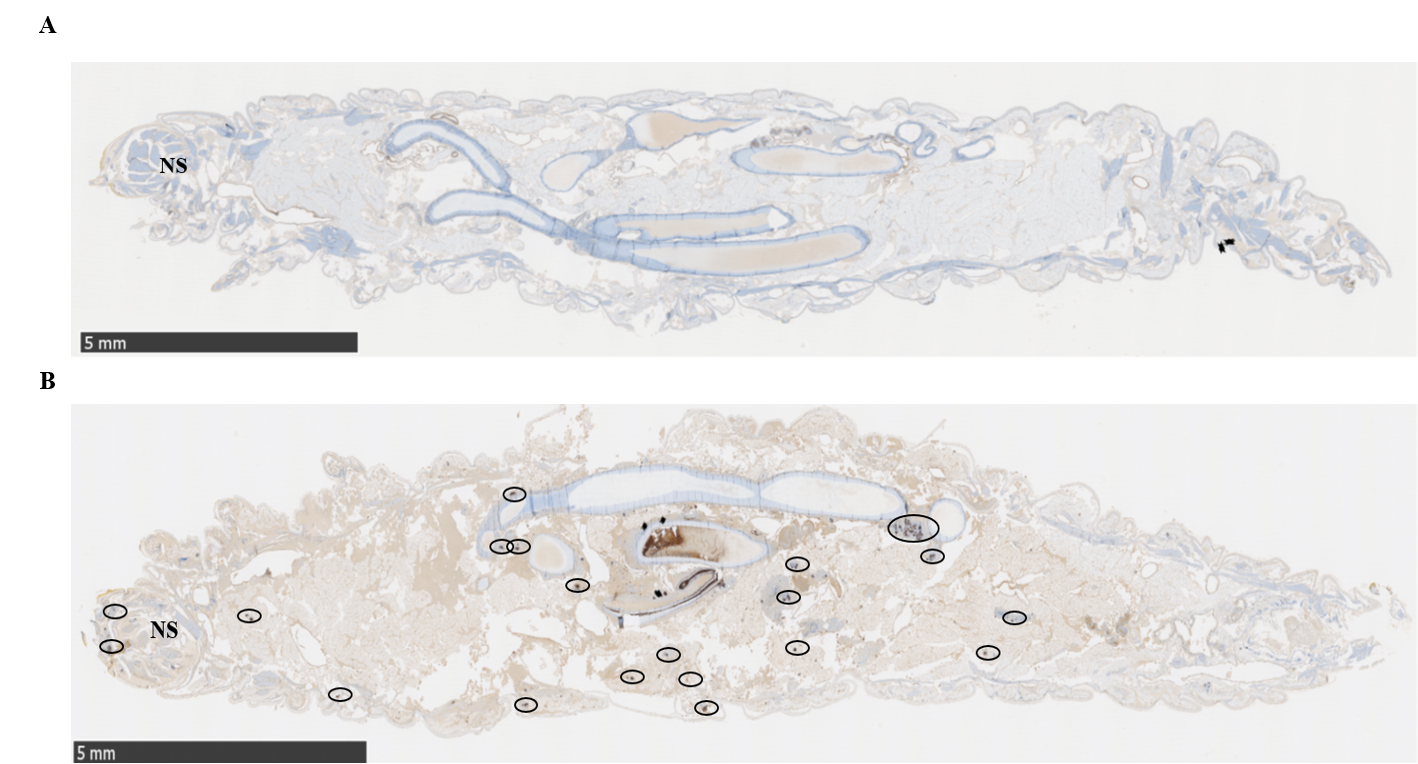

Supplement: Supplementary Figure 2 — Cross-sectional analysis for monitoring infection in the larvae. (A) PBS-injected larvae, the negative controls. (B) Larvae were injected with 106 CFU of S. aureus S75, and are shown here 96 hours later. Each black ellipse represents a nodule showing melanization, and the nodules are scattered around the entire larvae, including within the nervous system (NS). [file Image_2.tif]

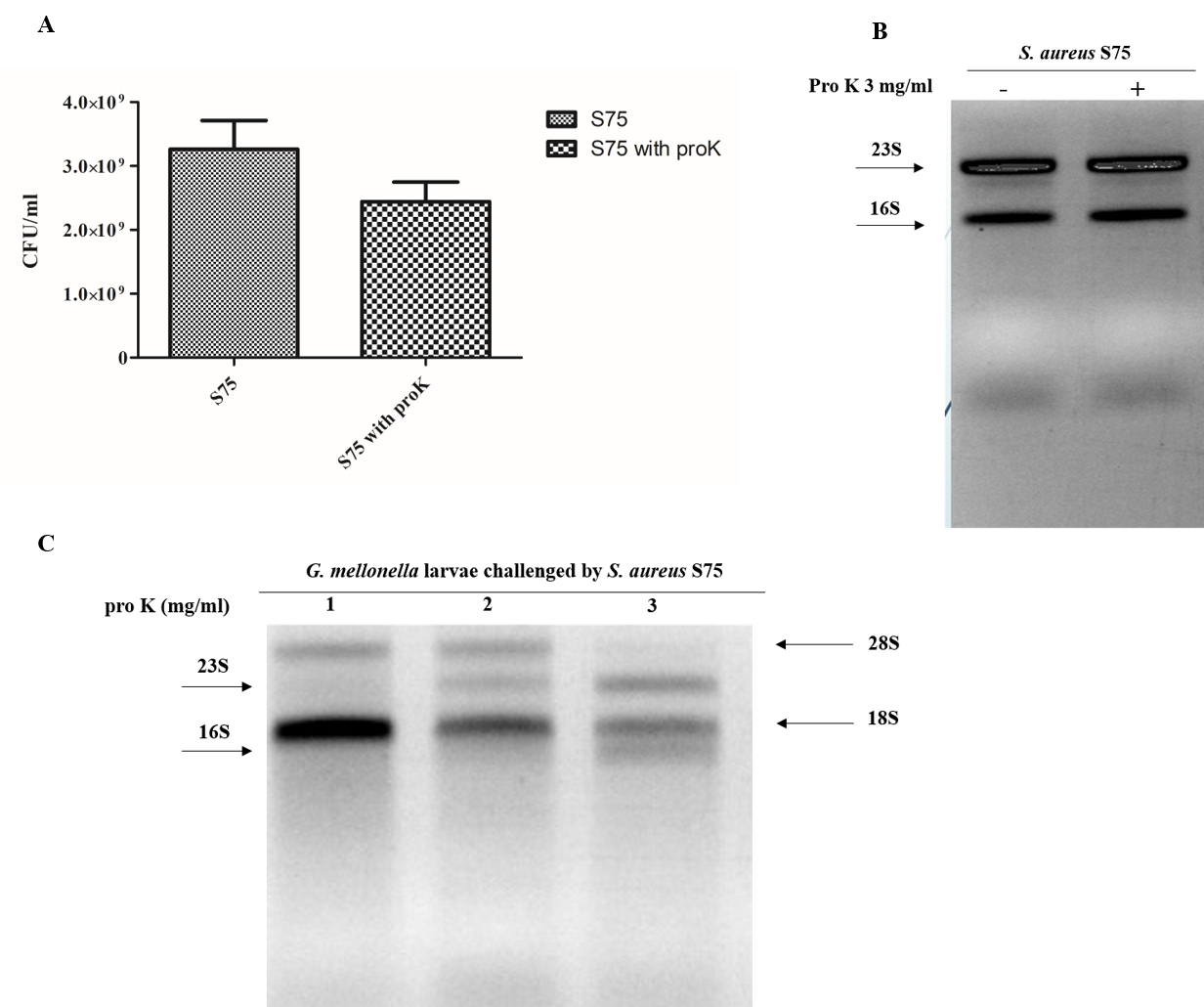

Supplement: Supplementary Figure 3 — Effect of Proteinase K on total RNA extractions. (A) Bacteria in the stationary phase were treated with 3 mg/ml Proteinase K (proK) or PBS for 30 minutes at 50°C. Results are the means of 3 independent experiments and are expressed in CFU/ml. (B) Total bacterial RNA counts after agarose gel electrophoresis and ethidium bromide exposure with (+) or without (-) a 3mg/ml Proteinase K solution. Bacterial RNA was extracted during the stationary phase after overnight culture. (C) Total RNA extraction of G. mellonella infected with 108 CFU of S. aureus S75. For each condition, 5 larvae were infected, and 2 were randomly chosen for RNA extraction (N= 6). RNA extraction was performed 30 minutes after injection, and several concentrations of Proteinase K were applied. For each condition, after DNAse process, total RNA quality and quantity were assessed using a NanoDrop spectrophotometer and 0.5% agarose gel electrophoresis. [file Image_3.tif]

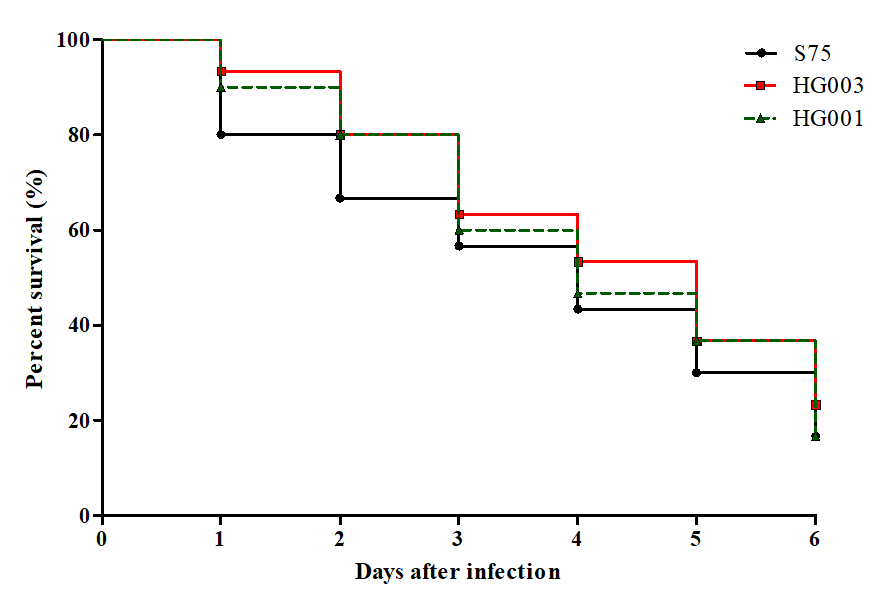

Supplement: Supplementary Figure 4 — Comparison of Kaplan-Meier survival plots of larvae infected with ST8 strains. Survival of G. mellonella larvae infected with 106 CFU of the S. aureus strains S75, HG003, and HG001. Mortality was monitored for 6 days. The plot is the result of 3 independent experiments using 10 larvae per group, and PBS-injected larvae were used as a negative control. [file Image_4.tif]

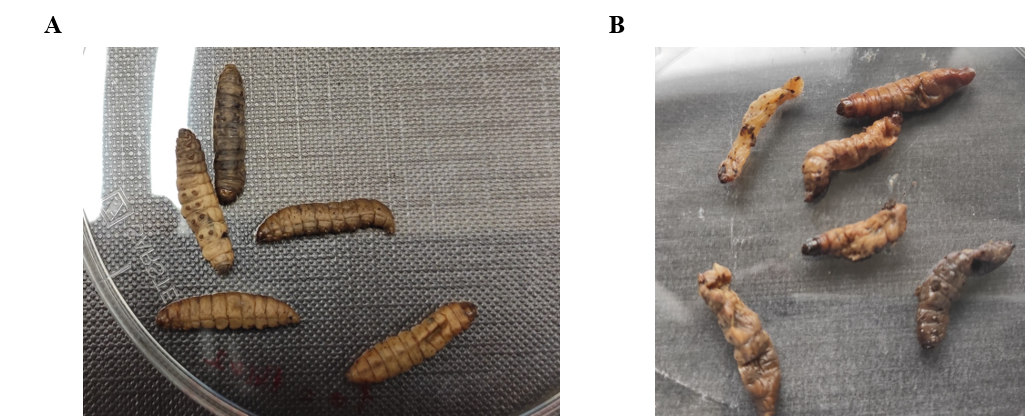

Supplement: Supplementary Figure 5 — RNAIII overexpression and macroscopic effects on infected G. mellonella larvae. Larvae were inoculated with 106 CFU and mortality monitored for 6 days, at which point dead larvae were observed. (A) Dead and melanized larvae after HG003-pRMC3 infection. (B) Larval necrosis was increased after infection with HG003-pRMC3-rnaIII. [file Image_5.tif]
