# Supplementary material for: Galleria mellonella Larvae as an Infection Model to Investigate sRNA-Mediated Pathogenesis in Staphylococcus aureus
Source: Front Cell Infect Microbiol. 2021 Apr 19;11:631710. doi: 10.3389/fcimb.2021.631710 (PMC8089379; doi:10.3389/fcimb.2021.631710)
Supplement: Supplementary Table 1 — Strains and vectors used for G. mellonella infection. [file Table_1.docx]

| **Strain / Vector** | **Relevant characteristics** | **References** |
| --- | --- | --- |
| *S. aureus* | | |
| S75 | methicillin-susceptible *S. aureus* strain isolated from bloodstream, t190 spa-type, Sequence Type 8 (ST8) | This study |
| Newman | methicillin sensible *S. aureus* strain isolated in 1952 from a human infection | [1] |
| HG003 | NCTC8325-derivative strain (*rsbU*^+^ and *tcaR*^+^) | [2] |
| HG003-*∆sprD* | HG003 strain deleted for *sprD* | This study |
| HG003-*∆rnaIII* | HG003 strain deleted for *rnaIII* | This study |
| HG001 | NCTC8325-derivative strain (*rsbU*^+^) | [2] |
| HG001-*∆sprC* | HG001 strain deleted for *sprC* | This study |
| HG001-*∆sprX* | HG001 strain deleted for *sprX* | This study |
| HG003-pRMC3 | HG003 with pRMC3 vector | This study |
| HG003-pRMC3-*rnaIII* | HG003 overexpressed RNAIII | This study |
| Plasmid | | |
| pRMC3 | pICS3-derivative vector | [3] |
| pRMC3-*rnaIII* | pRMC3, insertion vector carrying *rnaIII* under its native promoter; chlo^R^ (*S. aureus*) | This study |

References :

# Cheung AL, Projan SJ. Cloning and sequencing of sarA of *Staphylococcus aureus*, a gene required for the expression of agr. J Bacreriol. 1994;176(13); 4168–4172. doi: 10.1186/s12866-017-1129-9

# 2. Herbert S, Ziebandt A-K, Ohlsen K, Schäfer T, Hecker M, Albrecht D, et al. Repair of Global Regulators in *Staphylococcus aureus* 8325 and Comparative Analysis with Other Clinical Isolates. Infect Immun. 2010;78: 2877–2889. doi:10.1128/IAI.00088-10

3. Ivain L, Bordeau V, Eyraud A, Hallier M, Dreano S, Tattevin P, et al. An in vivo reporter assay for sRNA-directed gene control in Gram-positive bacteria: identifying a novel sRNA target in *Staphylococcus aureus*. Nucleic Acids Res 2017;45(8):4994-5007. doi: 10.1093/nar/gkx190
