# Supplementary material for: Galleria mellonella Larvae as an Infection Model to Investigate sRNA-Mediated Pathogenesis in Staphylococcus aureus
Source: Front Cell Infect Microbiol. 2021 Apr 19;11:631710. doi: 10.3389/fcimb.2021.631710 (PMC8089379; doi:10.3389/fcimb.2021.631710)
Supplement: Supplementary Table 2 — Primers used for qRTPCR. [file Table_2.docx]

| Target genes | Forward (5’-3’) | Reverse (5’-3’) | References |
| --- | --- | --- | --- |
| *S. aureus* | | | |
| *gyrB* | TTGACTTAAAAGAAGTTGGCACA | GCAAGCTCTCTAATACGCTGC | This study |
| *sigA* | GGATCAGGAAGCACAAAGTCC | TCTGCCGTCATCAAGACCAA | This study |
| *rnaIII* | AGATCACAGAGATGTGATGGAAA | TCTTGTGCCATTGAAATCACTCC | This study |
| *sprD* | ATGTATTGCTCCCCTTCGGG | AAGGTAAGCACCGAAATGCT | This study |
| *sprC* | TGCGTGGACAGTAAAACGAAG | TGAAGCTTCTACTCTCATGGCA | This study |
| *sprX* | TCACCCAAGCATGTCACTGG | ACTACGAGGGAGTAGTATGAAAAC | This study |
| *rsaA* | GCGACTGTATAATTTCTATTGAGGT | ACCCGAGTAGTCTTCCTTGG | This study |
