# Supplementary material for: Galleria mellonella Larvae as an Infection Model to Investigate sRNA-Mediated Pathogenesis in Staphylococcus aureus
Source: Front Cell Infect Microbiol. 2021 Apr 19;11:631710. doi: 10.3389/fcimb.2021.631710 (PMC8089379; doi:10.3389/fcimb.2021.631710)
Supplement: Supplementary Table 3 — Proteinase K Effects on the cycle thresholds of S. aureus housekeeping genes observed in qRTPCR. [file Table_3.docx]

|  | Target name | Ct mean | Ct SD |
| --- | --- | --- | --- |
| proK 1 mg/ml | *gyrB* | 21.64 | 0.33 |
|  | *sigA* | 18.66 | 0.10 |
| proK 2 mg/ml | *gyrB* | 17.51 | 0.34 |
|  | *sigA* | 15.14 | 0.27 |
| proK 3 mg/ml | *gyrB* | 14.64 | 0.01 |
|  | *sigA* | 11.37 | 0.14 |
